# Acknowledgment of Reviewers 2024

**DOI:** 10.1089/tmr.2024.87545.revack

**Published:** 2025-03-05

Critical evaluations of articles by expert reviewers make a vital contribution to ensuring the high quality of a journal’s content. The editorial leadership of *Telemedicine Reports* is very grateful for the support of the highly qualified peer reviewers who have dedicated their time and effort to reviewing our articles. We would like to show our appreciation by thanking the following individuals for their assistance with the review of articles for the journal in 2024.

Richard Albers

Ann Bagchi

Jordana Bernard

Kevin Chen

Linlin Chen

Ian Choe

Truong Van Dat

Christine Esper

Liliana Gehring

Suad Ghaddar

Scott Gorenstein

Heidi Greenberger

Judd Hollander

Sareh Keshvardoost

Elizabeth Krupinski

Maurice Mars

Intan Mohamad

Christine Mullins

Jonathan Neufeld

Jay Orlander

Nicolas Paul

Gabrielle Saunders

David Savage

Lee Schwamm

John Scott

Sanket Shah

Kimberly Shea

Yun Shen

Srinivasan Sridhar

Suemitsu Tokumassa

Kelsey Ufholz

